# A comparison of different diagnostic criteria of acute kidney injury in critically ill patients

**DOI:** 10.1186/cc13977

**Published:** 2014-07-08

**Authors:** Xuying Luo, Li Jiang, Bin Du, Ying Wen, Meiping Wang, Xiuming Xi

**Affiliations:** 1 Department of Critical Care Medicine, Fuxing Hospital, Capital Medical University, no. 20 Fuxingmenwai Street, Xicheng District, Beijing 100038, China; 2 Medical Intensive Care Unit, Peking Union Medical College Hospital, no. 1 Shuaifuyuan Wangfujing Dongcheng District, Beijing 100730, China

## Abstract

**Introduction:**

Recently, the Kidney Disease: Improving Global Outcomes (KDIGO) proposed a new definition and classification of acute kidney injury (AKI) on the basis of the RIFLE (Risk, Injury, Failure, Loss of kidney function, and End-stage renal failure) and AKIN (Acute Kidney Injury Network) criteria, but comparisons of the three criteria in critically ill patients are rare.

**Methods:**

We prospectively analyzed a clinical database of 3,107 adult patients who were consecutively admitted to one of 30 intensive care units of 28 tertiary hospitals in Beijing from 1 March to 31 August 2012. AKI was defined by the RIFLE, AKIN, and KDIGO criteria. Receiver operating curves were used to compare the predictive ability for mortality, and logistic regression analysis was used for the calculation of odds ratios and 95% confidence intervals.

**Results:**

The rates of incidence of AKI using the RIFLE, AKIN, and KDIGO criteria were 46.9%, 38.4%, and 51%, respectively. KDIGO identified more patients than did RIFLE (51% versus 46.9%, *P* = 0.001) and AKIN (51% versus 38.4%, *P* <0.001). Compared with patients without AKI, in-hospital mortality was significantly higher for those diagnosed as AKI by using the RIFLE (27.8% versus 7%, *P* <0.001), AKIN (32.2% versus 7.1%, *P* <0.001), and KDIGO (27.4% versus 5.6%, *P* <0.001) criteria, respectively. There was no difference in AKI-related mortality between RIFLE and KDIGO (27.8% versus 27.4%, *P* = 0.815), but there was significant difference between AKIN and KDIGO (32.2% versus 27.4%, *P* = 0.006). The areas under the receiver operator characteristic curve for in-hospital mortality were 0.738 (*P* <0.001) for RIFLE, 0.746 (*P* <0.001) for AKIN, and 0.757 (*P* <0.001) for KDIGO. KDIGO was more predictive than RIFLE for in-hospital mortality (*P* <0.001), but there was no difference between KDIGO and AKIN (*P* = 0.12).

**Conclusions:**

A higher incidence of AKI was diagnosed according to KDIGO criteria. Patients diagnosed as AKI had a significantly higher in-hospital mortality than non-AKI patients, no matter which criteria were used. Compared with the RIFLE criteria, KDIGO was more predictive for in-hospital mortality, but there was no significant difference between AKIN and KDIGO.

## Introduction

Acute kidney injury (AKI) is very common, especially in the intensive care unit (ICU). It is also associated with increased mortality and a longer stay in the hospital [1-7]. There have been many definitions, such as acute renal failure and renal impairment, and this has made it difficult to compare results across studies. In 2004, the Acute Dialysis Quality Initiative group proposed a classification for AKI: the Risk, Injury, Failure, Loss of Kidney Function, and End-stage Kidney Disease (RIFLE) classification, the first evidence-based consensus [8]. The classification includes three grades of severity of AKI (risk, injury, and failure) according to relative changes in serum creatinine (SCr) and urine output and two outcomes (loss of kidney function and end-stage kidney disease, or ESKD). It has been evaluated in many studies of critically ill patients with AKI and has shown good relevance for diagnosing and classifying the severity of AKI as well as comparable predictive ability for mortality [7,9-13].

In 2007, the Acute Kidney Injury Network (AKIN) group proposed a modified version of the RIFLE classification, which aimed to improve the sensitivity of AKI criteria [14]. There were several changes: an absolute increase in SCr of at least 26.4 μmol/L was added to stage 1; patients starting RRT were classified as stage 3, irrespectively of SCr; and the change in glomerular filtration rate (GFR) and the two outcome classes were removed. AKI diagnosis was based on change between two creatinine values within a 48-hour period for AKIN classification and within a 1-week window for RIFLE criteria. Severity of AKI in AKIN is staged over the course of 7 days by fold-change in creatinine from baseline.

The latest classification was proposed by the Kidney Disease: Improving Global Outcomes (KDIGO) Acute Kidney Injury Work Group, was based on the previous two classifications, and had the aim of unifying the definition of AKI [15]. According to this definition, AKI was diagnosed as an increase in SCr by at least 26.4 μmol/L within 48 hours or an increase in SCr to 1.5 times baseline, which is known or presumed to have occurred within 7 days before, or a urine volume of less than 0.5 mL/kg per hour for 6 hours. For KDIGO criteria, the 26.4 μmol/L increase needs to be within 48 hours but a 1.5-fold increase can occur within 7 days to diagnose AKI; and the 1-week or 48-hour timeframe is for diagnosis of AKI, not for staging. A patient can be staged over the entire episode of AKI. Increase in SCr to 3 times baseline, or SCr of more than 4.0 mg/dL (354 μmol/L), or starting RRT were all classified as stage 3. KDIGO removes the 0.5 mg/dL increase for creatinine more than 4 mg/dL to diagnose stage 3. Besides, KDIGO explicitly states that a rolling baseline can be used over 48-hour and 7-day periods for diagnosis of AKI, but it is unclear how this is handled in RIFLE or AKIN. The definition and difference among the three criteria are shown in Additional file 1.

Many studies have compared RIFLE with AKIN in critically ill patients, but only a few have compared KDIGO with these criteria in critically ill patients with AKI. The purposes of this study were to determine the incidence of AKI in critically ill patients according to the RIFLE, AKIN, and KDIGO criteria and to compare their predictive ability.

## Materials and methods

### Study cohort

This study used a database from a prospective, multicenter, observational study which investigated the epidemiology of AKI in critically ill patients at 30 ICUs of 28 tertiary hospitals in Beijing, China, from 1 March to 31 August 2012. (For a complete list of those hospitals and the persons responsible for the acquisition of data, see Additional file 2.) All patients who were older than 18 years and who were consecutively admitted to any participating ICU during the observational period were enrolled. For patients with multiple admissions, only the first admission was considered. Patients who had ESKD, underwent any renal replacement therapy (RRT), received kidney transplantation during the past 3 months, or stayed in the ICU for less than 24 hours were excluded.

### Data collection

Demographic data, dates of admission to the hospital and the ICU, primary diagnosis, co-morbidities, underlying chronic kidney disease, urine output (hourly or total urine volume in a 6-hour period), SCr, the need for mechanical ventilation, and the use of vasoactive drugs were continuously recorded for 10 days or until discharge from the ICU, whichever occurred earlier. Dates of discharge from the ICU and the hospital were also documented. In-hospital mortality was recorded as the primary outcome. Non-renal Sequential Organ Failure Assessment (SOFA) scores [16], Acute Physiology and Chronic Health Evaluation (APACHE) II score, and related clinical data were also recorded.

### Definition of acute kidney injury

The occurrence of AKI after ICU admission was determined by using the RIFLE, AKIN, and KDIGO criteria. Patients were categorized on the basis of SCr or urine output or both; the criteria that led to the worst classification were used. We did not use the GFR criteria. We used the lowest known SCr value during the past 3 months as the baseline creatinine in RIFLE and KDIGO criteria. For patients without known baseline, we used an estimated baseline or the lowest creatinine value during their stay in the ICU, whichever was lower. The baseline creatinine was estimated by using the simplified modification of diet in renal disease (MDRD) formula, assuming a GFR of 75 mL/min per 1.73 m^2^, and customized for the Chinese population, assuming a GFR of 75 mL/min per 1.73 m^2^[17]. In this study, the baseline creatinine of 754 patients was not known; the MDRD formula was applied for 120 patients to estimate baseline creatinine; for 634 patients, the lowest creatinine values during stay in the ICU were used as baseline. For AKIN criteria, the ICU admission creatinine was used as the baseline, and a rolling baseline was also used over the course of 48 hours. Severity of AKI based on AKIN is staged over the course of 7 days by change in creatinine. For KDIGO criteria, the 1-week or 48-hour timeframe was for diagnosis of AKI, not staging; and a patient can be staged over the entire episode of AKI. Patients were evaluated daily by using the RIFLE, AKIN, and KDIGO criteria after admission, until day 10 or discharge from the ICU, and the maximum RIFLE, AKIN, and KDIGO within ICU hospitalization were recorded. The worst classification during the patient’s ICU stay was used.

### Ethics

The study was approved by the institutional review boards of Fuxing Hospital, Capital Medical University, and all other participating hospitals (Additional file 3). The institutional review board specifically approved the informed consent waiver because of the anonymous and purely observational nature of this study.

### Statistical analysis

Data were analyzed by using SPSS 17.0.1 (SPSS Inc., Chicago, IL, USA). Non-normally distributed continuous variables were presented as median with interquartile range (IQR) and compared by Mann–Whitney *U* test or Kruskal-Wallis analysis-of-variance test with Bonferroni correction. The categorical data were reported as proportions and compared by using the Fisher exact test. Logistic regression analysis was used to assess the association of each RIFLE, AKIN, and KDIGO category with in-hospital mortality. ICU patients without AKI were used as the reference group. The discriminative ability of the criteria to correctly predict mortality was assessed by calculating the area under the curve (AUC) of the receiver operating characteristic (ROC) curve. A comparison of the ROC curves was performed by using a method described by DeLong and colleagues [18]. A *P* value of less than 0.05 was considered to be significant.

## Results

During the study period, 9,049 patients were consecutively admitted to one of 30 ICUs. In total, 5,942 patients were excluded; of these patients, 110 were younger than 18 years old, one received renal transplantation during the past 3 months, and 95 patients had received RRT before admission to the ICU. A further 5,725 patients were excluded because their length of stay in the ICU was less than 24 hours, and 11 were excluded because of insufficient clinical recordings. Finally, 3,107 patients were enrolled. The characteristics of the whole cohort are shown in Table 1.

**Table 1:** Characteristics of patients at baseline

**Characteristics**	**No-AKI**	**AKI by KDIGO**	**AKI missed by RIFLE**	**AKI missed by AKIN**
Age, median (IQR)	62 (49–74)	67 (53–78)	64 (53–75)	61 (48–74)
Male gender, n (%)	942 (61.9)	970 (61.2)	72 (57.1)	252 (64.5)
Surgical admission, n (%)	951 (62.4)	729 (46)	70 (55.6)	223 (57)
APACHE II score, median (IQR)	12 (8–16)	17 (12–23)	15 (11–22)	15 (10–21)
SOFA score, median (IQR)	4 (2–7)	6 (3–10)	6 (3–9)	6 (4–10)
Sepsis, n (%)	264 (17.3)	653 (41.2)	38 (30.2)	126 (32.2)
Mechanical ventilation, n (%)	957 (62.8)	1,132 (71.5)	101 (80.2)	275 (70.3)
Vasopressors, n (%)	466 (30.6)	864 (54.5)	67 (53.2)	201 (51.4)
Hypertension, n (%)	535 (35.1)	687 (43.4)	45 (35.7)	167 (42.7)
Diabetes, n (%)	212 (13.9)	320 (20.2)	23 (18.3)	74 (18.9)
Chronic heart failure, n (%)	65 (4.3)	152 (9.6)	8 (6.3)	23 (5.9)
Chronic liver disease, n (%)	38 (2.5)	53 (3.3)	5 (4)	6 (1.5)
Chronic lung disease, n (%)	68 (4.65)	98 (6.2)	8 (6.3)	24 (6.1)
Chronic kidney diseases, n (%)	36 (2.4)	167 (10.5)	13 (10.3)	24 (6.1)
Baseline SCr				
2-3 mg/dL, n (%)	5 (0.3)	30 (1.9)	4 (3.2)	3 (0.8)
3-4 mg/dL, n (%)	1 (0.1)	18 (1.1)	2 (1.6)	1 (0.3)
> 4 mg/dL, n (%)	3 (0.2)	27 (1.7)	2 (1.6)	4 (1)

AKI, acute kidney injury; AKIN, Acute Kidney Injury Network; APACHE II, Acute Physiology and Chronic Health Evaluation II; IQR, interquartile range; KDIGO, Kidney Disease: Improving Global Outcomes; RIFLE, Risk, Injury, Failure, Loss of Kidney Function, and End-stage Kidney Disease; SCr, serum creatinine; SOFA, Sequential Organ Failure Assessment.

### Comparison of incidence of acute kidney injury

AKI was diagnosed in 1,458 (46.9%) patients by using the RIFLE classification: 20.8% with Risk, 12.4% with Injury, and 13.8% with Failure. According to AKIN criteria, AKI occurred in 1,193 (38.4%) patients: 19% with stage 1, 6.6% with stage 2, and 12.8% with stage 3. When KDIGO criteria were used, AKI occurred in 1,584 (51%) patients: 23.1% with stage 1, 11.8% with stage 2, and 16% with stage 3. The KDIGO criteria were more sensitive than RIFLE (51% versus 46.9%, *P* <0.01) and AKIN (51% versus 38.4%, *P* <0.001).

A total of 259 patients received RRT within 10 days after ICU admission. According to the KDIGO and AKIN criteria, 247 of them were identified as AKI with stage 3; the other 12 patients without AKI received RRT for a number of reasons, including sepsis and drug overdose. On the basis of the RIFLE criteria, 245 patients were diagnosed with AKI: 14 with Risk, 33 with Injury, and 198 with Failure.

The KDIGO criteria identified 126 more patients with AKI than the RIFLE criteria did: 106 with stage 1, 12 with stage 2, and 8 with stage 3 (Table 2). Among them, 124 patients were identified by an increase in creatinine alone, and the other two patients received RRT. Seventy patients were defined by KDIGO as stage 3 but not as failure by RIFLE (19 with Risk, 44 with Injury, and 8 without AKI), and 49 of them received RRT.

**Table 2:** Agreement between RIFLE and KDIGO classifications

**Definition**		**RIFLE**				
		**No-AKI**	**Risk**	**Injury**	**Failure**	**Total**
KDIGO	No-AKI	1,523 (49)	0	0	0	1,523 (49)
	Stage 1	106 (3.4)	612 (19.7)	0	0	718 (23.1)
	Stage 2	12 (0.4)	15 (0.5)	341 (11)	0	368 (11.8)
	Stage 3	8 (0.3)	18 (0.6)	44 (1.4)	428 (13.8)	498 (16)
	Total	1,649 (53.1)	645 (20.8)	385 (12.4)	428 (13.8)	3,107 (100)

AKI, acute kidney injury; KDIGO, Kidney Disease: Improving Global Outcomes; RIFLE, Risk, Injury, Failure, Loss of Kidney Function, and End-stage Kidney Disease.

Compared with the AKIN criteria, KDIGO diagnosed 391 more patients as having AKI; 270 of them were categorized as stage 1, 84 as stage 2, and 37 as stage 3 (Table 3). Among 391 patients, only 25 patients had chronic kidney disease. However, the median creatinine of these 391 patients on the first day of ICU admission was 118.6 μmol/L (IQR 78 to 159.7), which was much higher than the baseline: 118.6 (IQR 78 to 159.7) versus 70 (IQR 49 to 86), *P* <0.001.

**Table 3:** Agreement between AKIN and KDIGO classifications

**Definition**		**AKIN**				
		**No-AKI**	**Stage 1**	**Stage 2**	**Stage 3**	**Total**
KDIGO	No-AKI	1,523 (49)	0	0	0	1,523 (49)
	Stage 1	270 (8.7)	448 (14.4)	0	0	718 (23.1)
	Stage 2	84 (2.7)	100 (3.2)	184 (5.9)	0	368 (11.8)
	Stage 3	37 (1.2)	43 (1.4)	21 (0.7)	397 (12.8)	498 (16)
	Total	1,914 (61.6)	591 (19)	205 (6.6)	397 (12.8)	3,107 (100)

AKI, acute kidney injury; AKIN, Acute Kidney Injury Network; KDIGO, Kidney Disease: Improving Global Outcomes.

### Comparison of outcomes

#### In-hospital mortality

Crude in-hospital mortality was significantly higher for AKI patients than for non-AKI patients, regardless of the definition used: the RIFLE (27.8% versus 7%, *P* < 0.0001), AKIN (32.2% versus 7.1%, *P* < 0.0001) and KDIGO (27.4% versus 5.6%, *P* < 0.0001) criteria. Mortality rate of patients identified as AKI by AKIN was higher than by KDIGO or RIFLE (32.2% versus 27.4%, *P* = 0.006, and 32.2% versus 27.8%, *P* = 0.013; respectively) but did not differ significantly between RIFLE and KDIGO (27.8% versus 27.4%, *P* = 0.82) (Table 4).

**Table 4:** In-hospital mortality according to AKI stratified by the RIFLE, AKIN, and KDIGO classification schemes

**Category**	**RIFLE**	**AKIN**	**KDIGO**
None (%)	115 (7)	136 (7.1)	86 (5.6)
Risk/Stage 1 (%)	102 (15.8)	126 (21.3)	111 (15.5)
Injury/Stage 2 (%)	111 (28.8)	69 (33.7)	103 (28)
Failure/Stage 3 (%)	192 (44.9)	189 (47.6)	220 (44.2)
Any category (%)	405 (27.8)	384 (32.2)	434 (27.4)

AKI, acute kidney injury; AKIN, Acute Kidney Injury Network; KDIGO, Kidney Disease: Improving Global Outcomes; RIFLE, Risk, Injury, Failure, Loss of Kidney Function, and End-stage Kidney Disease.

We also compared the in-hospital mortality of patients without AKI according different criteria and found that the patients identified by KDIGO but missed by AKIN or RIFLE had higher mortality than patients with no-AKI based on KDIGO (12.8% versus 5.6%, *P* < 0.01; 23% versus 5.6%, *P* < 0.001).

The mortality rates of patients missed by the RIFLE criteria but identified by KDIGO as stage 1, stage 2, and stage 3 were 20.8%, 33.3%, and 37.5%, respectively. The mortality rates of those missed by the AKIN criteria but identified by KDIGO as stage 1, stage 2, and stage 3 were 9.6%, 19%, and 21.6%, respectively.

### Length of intensive care unit stays (alive)

In our study, length of ICU stay was longer in patients with AKI than in those without AKI, no matter which criteria were used: the RIFLE (5 [3-10] versus 3 [2-6], *P* < 0.001), AKIN (5 [3-11] versus 3 [2-6], *P* < 0.001), and KDIGO (5 [3-10] versus 3 [2-6], *P* < 0.001) criteria. For patients missed by RIFLE or AKIN but identified by KDIGO, length of ICU stay was also longer than that of patients with no-AKI based on KDIGO (5 [3-8] versus 3 [2-6], *P* < 0.01; [3-10] versus 3 [2-6], *P* < 0.01; respectively).

### Predictive ability for mortality

Irrespectively of which definition was used, AKI was independently associated with in-hospital mortality even after adjustment for age, gender, diabetes, hypertension, chronic kidney disease, chronic heart failure, and SOFA score (without renal component) (Table 5).

**Table 5:** Association of different acute kidney injury category with mortality by multivariable logistic regression models

**Criteria**	**Odds ratio (95% CI)**	***P *** **value**
RIFLE		
Risk	1.96 (1.46-2.64)	< 0.001
Injury	3.48 (2.55-4.75)	< 0.001
Failure	6.95 (5.19-9.30)	< 0.001
AKIN		
Stage 1	2.62 (1.99-3.45)	< 0.001
Stage 2	4.63 (3.22-6.65)	< 0.001
Stage 3	7.75 (5.82-10.32)	< 0.001
KDIGO		
Stage 1	2.38 (1.75-3.23)	< 0.001
Stage 2	4.31 (3.09-6.02)	< 0.001
Stage 3	8.54 (6.31-11.56)	< 0.001

The model is adjusted for age, gender, diabetes, hypertension, chronic kidney disease, chronic heart failure, and Sequential Organ Failure Assessment (SOFA) score (without renal component). AKI, acute kidney injury; AKIN, Acute Kidney Injury Network; CI, confidence interval; KDIGO, Kidney Disease: Improving Global Outcomes; RIFLE, Risk, Injury, Failure, Loss of Kidney Function, and End-stage Kidney Disease.

For patients diagnosed as AKI by KDIGO but not by RIFLE, AKI was also an independent risk factor of in-hospital mortality (odds ratio (OR) 4.498, 95% confidence interval (CI) 3.727 to 5.429, *P* < 0.001) even after adjustment for age, gender, diabetes, hypertension, chronic kidney disease, chronic heart failure, and SOFA score (without renal component). Similarly, for patients identified as AKI by KDGIO but not by AKIN, AKI was an independent risk factor for mortality (OR 1.963, 95% CI 1.139 to 2.898, *P* < 0.01).

**Figure 1:**
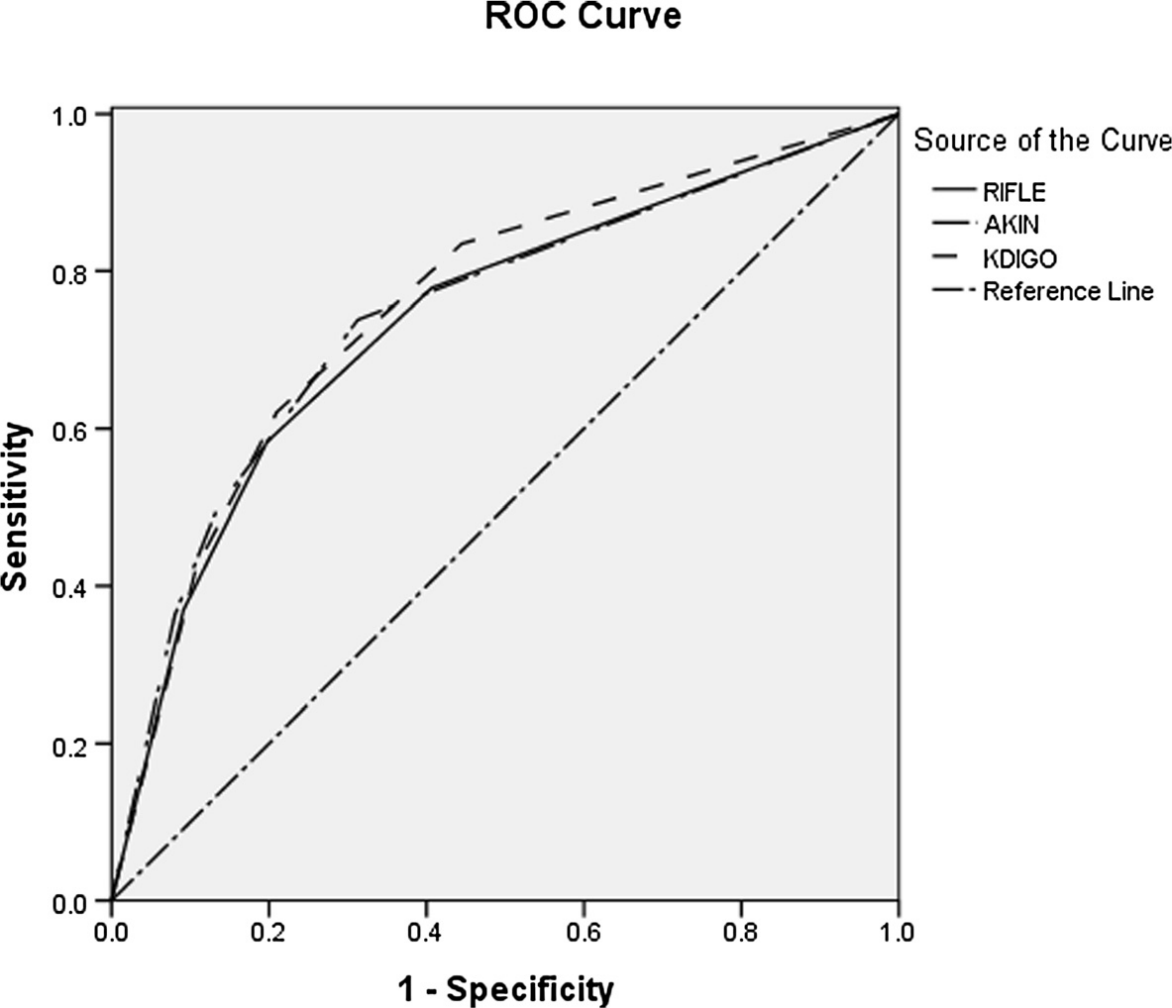
**Area under the curves for RIFLE, AKIN, and KDIGO classification schemes comparing the predictive ability of RIFLE, AKIN, and KDIGO classification schemes for in-hospital mortality.** AKIN, Acute Kidney Injury Network; KDIGO, Kidney Disease: Improving Global Outcomes; RIFLE, Risk, Injury, Failure, Loss of Kidney Function, and End-stage Kidney Disease; ROC, receiver operating characteristic. RIFLE: Area Under the Curve 0.738 (95% CI 0.713-0.762, P < 0.001). AKIN: Area Under the Curve 0.746 (95% CI 0.721-0.770, P < 0.001). KDIGO: Area Under the Curve 0.757 (95% CI 0.733-0.780, P < 0.001.

The area-under-ROC curves for in-hospital mortality for RIFLE, AKIN, and KDIGO criteria were 0.738 (*P* < 0.001), 0.746 (*P* < 0.001), and 0.757 (*P* < 0.001), respectively. Compared with the RIFLE criteria, KDIGO had greater predictive ability for in-hospital mortality (*P* < 0.001) (Figure 1 and Table 6). But there was no significant difference between AKIN and KDIGO (*P* = 0.38).

**Table 6:** Predictive ability of RIFLE, AKIN, and KDIGO for in-hospital mortality

**Predictive factors**	**Cutoff point**	**Sensitivity, %**	**Specificity, %**	**+LR**	**-LR**
RIFLE	Risk-Failure	77.9	59.3	1.91	0.37
	Injury-Failure^a^	58.3	80.3	2.96	0.52
	Failure	36.9	90.0	4.05	0.69
AKIN	Stage 1-Stage 3^a^	73.9	68.7	2.36	0.38
	Stage 2-Stage 3	49.6	86.7	3.73	0.58
	Stage 3	36.4	92.0	4.52	0.69
KDIGO	Stage 1-Stage 3	83.5	55.6	1.88	0.30
	Stage 2-Stage 3^a^	62.1	79.0	2.96	0.48
	Stage 3	42.3	89.3	3.94	0.65

^a^Value is the best cutoff point. AKIN, Acute Kidney Injury Network; KDIGO, Kidney Disease: Improving Global Outcomes; +LR, positive likelihood ratio; -LR, negative likelihood ratio; RIFLE, Risk, Injury, Failure, Loss of Kidney Function, and End-stage Kidney Disease.

### Patients with known baseline

For patients with known baseline (n = 2,353), the rates of incidence of AKI according to RIFLE, AKIN, and KDIGO were 45.5%, 39%, and 50.6%, respectively. The KDIGO criteria were more sensitive than RIFLE (50.6% versus 45.5%, *P* < 0.01) and AKIN (50.6% versus 39%, *P* < 0.001). Compared with patients without AKI, in-hospital mortality was significantly higher for those diagnosed as AKI by the RIFLE (27.8% versus 7.3%, *P* < 0.001), AKIN (31.7% versus 7%, *P* < 0.001), and KDIGO (27.4% versus 5.7%, *P* < 0.001) criteria. There was no difference in AKI-related mortality between RIFLE and KDIGO (*P* = 0.82), but there was significant difference between AKIN and KDIGO (31.7% versus 27.4%, *P* =0.031). These results were identical to that of the whole study cohort.

## Discussion

Numerous studies have compared the RIFLE and AKIN criteria for AKI. However, the incidence of AKI still varied. Based on these two criteria, the KDIGO criteria were recently proposed in order to unify the definition of AKI. To date, only a few previous studies have compared the incidence and mortality of AKI in critically ill patients according to these three definitions [19-21]. This is the first, large, multicenter study to compare these three different criteria in critically ill patients with AKI in China.

The incidence of AKI according to the KDIGO criteria was higher than that defined by RIFLE and AKIN, even after we excluded patients without known baseline creatinine. It was similar to the results of a study comparing definitions of AKI in hospitalized individuals in Boston [20] but differed from a retrospective study of patients after cardiac surgery, which concluded that incidence and outcome of AKI according to the RIFLE, AKIN, and KDIGO classification were similar [19]. The study of hospitalized patients conducted by Fujii and colleagues in Japan concluded that the rates of incidence of AKI according to RIFLE, AKIN, and KDIGO were 11%, 4.8%, and 11.6%, respectively [21]. KDIGO classified 126 (4.1%) more patients with AKI than RIFLE did, the majority of which were patients with stage 1. An in-depth analysis of these patients found that the majority (124 patients) were identified by a small increase in creatinine alone but that the remaining two patients received RRT at the same time. Firstly, we found that for some patients there was a decrease in creatinine after admission to the ICU, followed by a relative increase; these patients could be identified by KDIGO and AKIN because a rolling in-hospital baseline was used for the 48-hour rise, but not by RIFLE. Secondly, patients who received RRT would be classified as stage 3 by KDIGO and AKIN, irrespectively of SCr, but not by RIFLE.

When compared with AKIN, KDIGO diagnosed AKI in an additional 391 patients, including 25 patients with chronic kidney disease; these patients were predominantly stage 1, followed by stage 2 and stage 3. The median creatinine level in these 391 patients on their first day of admission to the ICU was much higher than the baseline level, and this means that AKI may have been present on the day of ICU admission or even before. According to the AKIN criteria, AKI was diagnosed by two creatinine measurements within 48 hours. However, most patients did not have creatinine measured every day prior to the ICU admission: thus, when creatinine at ICU admission was used, some community-acquired AKI cases may have been missed [22-24]. In addition, patients with a slow reduction of renal function may have been missed by the AKIN criteria [25]. The KDIGO definition reserved the baseline creatinine from RIFLE as well as a small increase in creatinine from AKIN criteria, allowing greater sensitivity than RIFLE and AKIN.

All definitions showed comparable and excellent associations with worse outcome according to increased severity of AKI. As for the predictive ability of these criteria, all were found to be significant predictors of increased mortality using multivariate analysis adjusting for age, gender, diabetes, hypertension, chronic kidney disease, chronic heart failure, and SOFA score. These findings were identical to those of previous studies [3,4,26,27]. Patients missed by RIFLE but identified by KDIGO, most of which were classified as stage 1, had a longer length of ICU stay than no-AKI patients based on KDIGO. The patients diagnosed by KDIGO criteria as stage 1 but missed by RIFLE had much higher mortality than patients without AKI based on KDIGO (20.8% versus 5.6%, *P* < 0.001). Thus, we deduced that a small increase in creatinine might be accompanied by increased mortality. Similar results were observed in other studies [28,29]. A study by Wilson and colleagues determined that the magnitude of the decrease in creatinine generation rate may be correlated with the severity of illness [30]. In other words, the patients with a small increase in creatinine, accompanied by increased mortality and longer hospital stay, could be identified by KDIGO but not by RIFLE. The KDIGO definitions also showed a little better predictive ability than RIFLE did, according to the AUC curve for in-hospital mortality. For patients missed by AKIN but not by KDIGO, AKI was also an independent risk factor for mortality, but of low risk; and the mortality of these patients was only a little higher than that of no-AKI patients according to the KDIGO criteria (12.8% versus 5.6%, *P* < 0.01). In addition, the mortality of patients with AKI based on AKIN was a little higher than those on KDIGO (32.2% versus 27.4%, *P* = 0.006) and this was probably because KDIGO identified more patients in a mild severity level of AKI, with a relatively low mortality rate. According to the AUC curve, there was no significant difference between KDIGO and AKIN in the predictive ability for in-hospital mortality (0.757 versus 0.746, *P* = 0.12). Therefore, we concluded that KDIGO and AKIN were comparable on their predictive ability for in-hospital mortality. So whether this small increase in the mortality of these patients, identified by KDIGO but missed by AKIN, is of high risk requires more research. However, the study of hospitalized patients in Japan concluded that KDIGO and RIFLE achieved similar discrimination but that the discrimination of AKIN was inferior [21]. Given that their conclusion is different from ours, maybe more study is needed.

There are some limitations to our study. First, we used the simplified MDRD formula as baseline for patients without known baseline creatinine. In a prospective observational study, a good correlation of estimated as compared with observed baseline values was found for patients without chronic kidney disease [31]. Second, we did not have any records of creatinine during hospitalization but we did have records prior to ICU admission, and this may have caused the incidence of AKI by AKIN to be underestimated. The AKIN criteria recommend applying only the urine output criteria “following adequate fluid resuscitation”, which is ambiguous. In our study, we did not adhere strictly to this recommendation. Third, we received hourly records of urine output for most patients, but for others only the total urine volume in a 6-hour period was recorded. A study by Etienne Macedo and colleagues [32] concluded that there was no significant difference between assessing urine output every hour or the total urine volume in a 6-hour period for the detection of episodes of oliguria, and the latter did not decrease their sensitivity for identifying patients with AKI. Finally, we did not have data regarding additional factors that could influence urine output, such as diuretic therapy.

## Conclusions

The incidence of AKI in critically ill patients varied according to the criteria used. The KDIGO criteria identified more patients as AKI than RIFLE and AKIN did. Compared with the RIFLE criteria, KDIGO was more predictive for in-hospital mortality, but there was no significant difference between AKIN and KDIGO.

## Key messages

• KDIGO identified more patients as AKI than RIFLE and AKIN did.

• AKI was independently associated with in-hospital mortality, irrespectively of which definition was used.

• For the patients diagnosed as AKI by KDIGO but not by RIFLE or AKIN, AKI was also an independent risk factor of mortality.

• KDIGO was more predictive for in-hospital mortality than RIFLE was.

## Abbreviations

AKI: acute kidney injury; AKIN: Acute Kidney Injury Network; AUC: area under the curve; CI: confidence interval; ESKD: end-stage kidney disease; GFR: glomerular filtration rate; ICU: intensive care unit; IQR: interquartile range; KDIGO: Kidney Disease: Improving Global Outcomes; MDRD: simplified modification of diet in renal disease; OR: odds ratio; RIFLE: Risk, Injury, Failure, Loss of Kidney Function, and End-stage Kidney Disease; ROC: receiver operating characteristic; RRT: renal replacement therapy; SCr: serum creatinine; SOFA: Sequential Organ Failure Assessment.

## Competing interests

The authors declare that they have no competing interests.

## Authors’ contributions

XL and LJ designed and carried out the study, performed the statistical analysis, and drafted the manuscript. BD was involved in design and in acquisition of data and helped to revise the manuscript critically for important content. YW and MW were involved in the design and the statistical analysis. The Beijing Acute Kidney Injury Trial (BAKIT) Workgroup participated in acquisition and interpretation of data. XX conceived of the study, participated in its design, and helped to revise manuscript. All authors read and approved the final manuscript.

## Supplementary Material

Additional file 1 **RIFLE, AKIN, and KDIGO criteria for AKI.** The definition and difference among these three criteria are shown in detail. AKI, acute kidney injury; AKIN, Acute Kidney Injury Network; ESKD, end-stage kidney disease; GFR, glomerular filtration rate; KDIGO, Kidney Disease: Improving Global Outcomes; RIFLE, Risk, Injury, Failure, Loss of Kidney Function, and End-stage Kidney Disease; RRT, renal replacement therapy; Scr, serum creatinine. Click here for file

Additional file 2 Members of the Beijing Acute Kidney Injury Trial (BAKIT) workgroup. Click here for file

Additional file 3 All other ethical bodies that approved our study in the various centers involved. Click here for file
