# Supplementary material for: A comparison of different diagnostic criteria of acute kidney injury in critically ill patients
Source: Crit Care. 2014 Jul 8;18(4):R144. doi: 10.1186/cc13977 (PMC4227114; doi:10.1186/cc13977)
Supplement: Additional file 1 — RIFLE, AKIN, and KDIGO criteria for AKI. The definition and difference among these three criteria are shown in detail. AKI, acute kidney injury; AKIN, Acute Kidney Injury Network; ESKD, end-stage kidney disease; GFR, glomerular filtration rate; KDIGO, Kidney Disease: Improving Global Outcomes; RIFLE, Risk, Injury, Failure, Loss of Kidney Function, and End-stage Kidney Disease; RRT, renal replacement therapy; Scr, serum creatinine. [file cc13977-S1.docx]

**Additional file 1**

**RIFLE, AKIN and KDIGO criteria for AKI**

|  | Serum creatinie criteria | Urine output criteria |
| --- | --- | --- |
| RIFLE [8] | Increase in SCr ≥ 50% within 7 d |  |
| Risk | Increase in SCr≥ 1.5 x baseline  Or GFR decease > 25% | <0.5ml/kg/h  for ≥6h |
| Injury | Increase in SCr≥2 x baseline  Or GFR decease > 50% | <0.5ml/kg/h for≥12 h |
| Failure | Increase in SCr ≥3x baseline  Or SCr≥354µmol/L with an acute rise of at least 44µmol/L or GFR decease > 75% | <0.3ml/kg/h≥24h or anuria≥12 |
| Loss | Complete loss of kidney function > 4wk |  |
| ESKD | Complete loss of kidney function > 3mo |  |
| AKIN [14] | Increase in SCr ≥26.4µmol/L or ≥ 50% within 48h |  |
| Stage 1 | Increase in SCr≥26.4µmol/L  Or increase ≥1.5 x baseline | <0.5ml/kg/h  for ≥6h |
| Stage 2 | Increase in SCr ≥2 x baseline | <0.5ml/kg/h for≥12 h |
| Stage 3 | Increase in SCr ≥3 x baseline, or SCr ≥ 354µmol/L with an acute rise of at least 44µmol/L  Or initiation of RRT | <0.3ml/kg/h≥24h or anuria≥12h |
| KDIGO [15] | Increase in SCr≥26.4µmol/L within 48h  Or ≥50% within 7 d |  |
| Stage 1 | Increase in SCr≥26.4µmol/L in 48 h  Or increase ≥1.5 x baseline | <0.5ml/kg/h  for ≥6h |
| Stage 2 | Increase in SCr ≥2 x baseline | <0.5ml/kg/h for≥12 h |
| Stage 3 | Increase in SCr ≥3 x baseline  Or increase in SCr to ≥354µmol/L  Or initiation of RRT irrespective of SCr  Or in patients<18 years, decrease in eGFR to<35 ml/min per 1.73 m^2^ | <0.3ml/kg/h≥24h or anuria≥12 |

ESKD indicates end-stage kidney disease; GFR, glomerular filtration rate; RRT, renal replacement therapy; Scr, Serum creatinie.
